# Leber congenital amaurosis/early-onset severe retinal dystrophy: current management and clinical trials

**DOI:** 10.1136/bjophthalmol-2020-318483

**Published:** 2021-03-12

**Authors:** Malena Daich Varela, Thales Antonio Cabral de Guimaraes, Michalis Georgiou, Michel Michaelides

**Affiliations:** 1 UCL Institute of Ophthalmology, University College London, London, UK; 2 Moorfields Eye Hospital NHS Foundation Trust, London, London, UK

**Keywords:** genetics, retina, dystrophy, degeneration

## Abstract

Leber congenital amaurosis (LCA) is a severe congenital/early-onset retinal dystrophy. Given its monogenic nature and the immunological and anatomical privileges of the eye, LCA has been particularly targeted by cutting-edge research. In this review, we describe the current management of LCA, and highlight the clinical trials that are on-going and planned. *RPE65*-related LCA pivotal trials, which culminated in the first Food and Drug Administration-approved and European Medicines Agency-approved ocular gene therapy, have paved the way for a new era of genetic treatments in ophthalmology. At present, multiple clinical trials are available worldwide applying different techniques, aiming to achieve better outcomes and include more genes and variants. Genetic therapy is not only implementing gene supplementation by the use of adeno-associated viral vectors, but also clustered regularly interspaced short palindromic repeats (CRISPR)-mediated gene editing and post-transcriptional regulation through antisense oligonucleotides. Pharmacological approaches intending to decrease photoreceptor degeneration by supplementing 11-*cis*-retinal and cell therapy’s aim to replace the retinal pigment epithelium, providing a trophic and metabolic retinal structure, are also under investigation. Furthermore, optoelectric devices and optogenetics are also an option for patients with residual visual pathway. After more than 10 years since the first patient with LCA received gene therapy, we also discuss future challenges, such as the overlap between different techniques and the long-term durability of efficacy. The next 5 years are likely to be key to whether genetic therapies will achieve their full promise, and whether stem cell/cellular therapies will break through into clinical trial evaluation.

## Introduction

Leber congenital amaurosis (LCA) represents one of the severest diagnoses a family can receive regarding a child’s eyesight. It is characterised by early-onset visual impairment, nystagmus or roving eyes and severe photoreceptor dysfunction.^1^ LCA and early-onset severe retinal degeneration (EOSRD) belong to the same disease spectrum, with the latter being less severe; later onset of visual impairment, often better preserved visual acuity and decreased, but usually present electrophysiological responses.^1^ LCA/EOSRD affect approximately 1 in 80 000 children worldwide.^2^ Retinal examination can be unremarkable especially in the early stages or show mild signs such as retinal pigment epithelial mottling and vessel narrowing.^3^ Salt and pepper retinopathy, optic disc pallor and retinal pigment clumping (including nummular and bone spicule) usually appear in older individuals.

**Figure 1:**
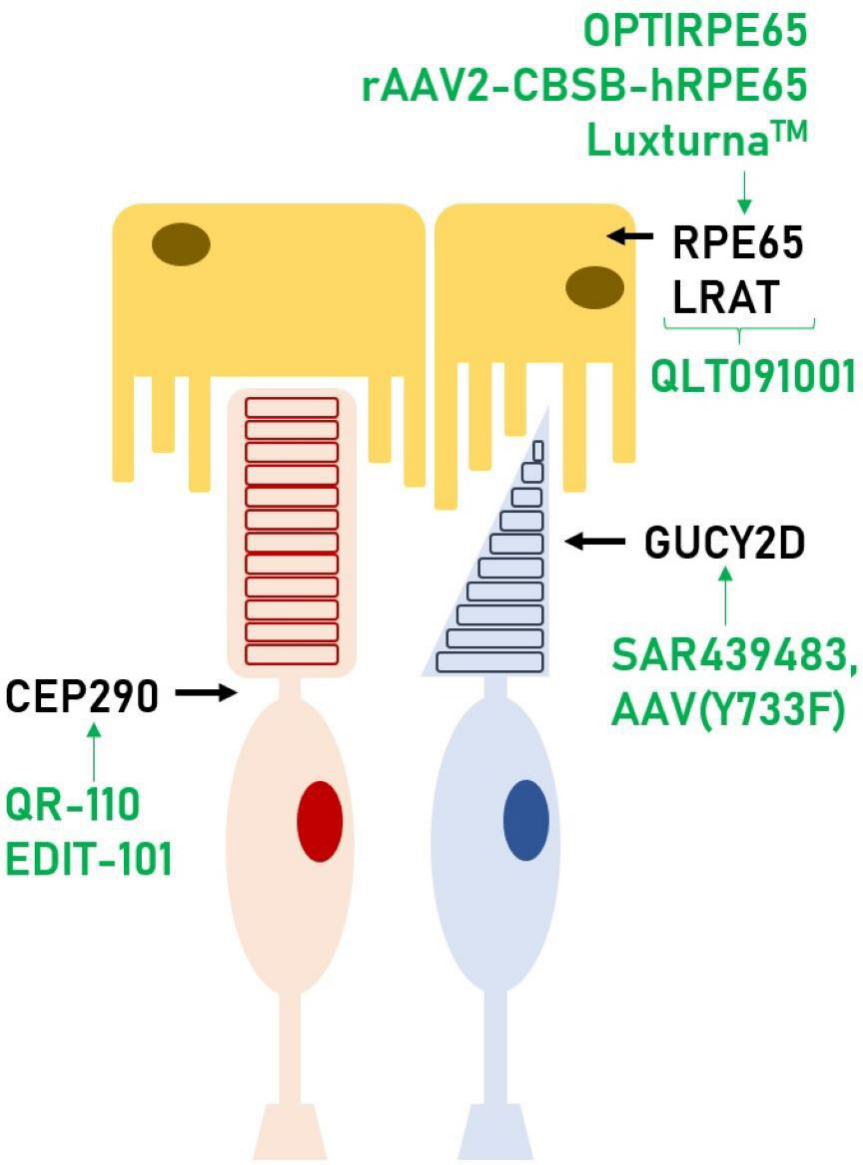
Photoreceptor (rod in red, cone in blue) and RPE cells. In this scheme, we can see where each gene discussed in this review has its function within the retina. These genes are present in both rods and cones, however, we depicted it showing the cell type where relative expression is highest. Also, in green, the therapies that are currently available or under investigation for those genes. RPE, retinal pigment epithelium.

The diagnosis of LCA is clinical; by ophthalmological evaluation and electrophysiology. However, genetic testing is critical to confirm the specific diagnosis and is key towards the administration of genotype-specific treatment, inclusion in clinical trials, prognostication, as well as for family planning and tailored screening of systemic conditions.^4^ LCA can be an isolated eye condition or part of a syndrome, such as Senior Loken or Joubert syndrome.^5 6^ Moreover, it is genetically heterogeneous and can be caused by variants in at least 25 genes such as *RPE65*, *GUCY2D*, *NMNAT1*, *CEP290*, *AIPL1*, and *RDH12*.^1^ Some of these genotypes are associated with distinctive phenotypic features including, *RDH12*, *CRX*, *CRB1* and *NMNAT1* can present with early-onset macular atrophy,^7^ *CEP290* may be associated with other ciliopathy manifestations,^8^ *RPE65* may show absent or decreased autofluorescence.^9^ Furthermore, the pathophysiology and location where each faulty gene has its repercussion(s) is also different. AIPL1 is predominantly located in rod outer segments and reduces the intracellular concentration of cyclic guanosine monophosphate (cGMP).^10^ CRB1 is located in the cell membrane of Müller cells and has a role in protein scaffold.^11^ LRAT and RPE65 are located in retinal pigment epithelium (RPE) cells and are involved in the visual cycle.^12^ The retinal location of the proteins encoded by the genes that will be discussed in this review are depicted in figure 1.

Due to the severe visual impairment caused by LCA/EOSRD, the well-established genetic basis, availability of animal models and well-characterised clinical features, translational research has been particularly active. In this review, we aim to provide an update of the treatments that are currently available and those that are in clinical trial (table 1).

**Table 1:** Summary of ongoing clinical trials targeting LCA/EOSRD

Gene	Drug	Clinical trial ID (NCT #)	Study phase	Route of delivery	Status	Sponsor	Location	Number of participants
*RPE65*	rAAV 2/2.hRPE65p.hRPE65–OPTIRPE65	NCT00643747– NCT02781480	Phase I–II	Subretinal	Completed	MeiraGTx	UK	12–15
*RPE65*	rAAV2-CBSB–hRPE65	NCT00481546	Phase I	Subretinal	Active, not recruiting	University of Pennsylvania	USA	15
*RPE65*	voretigene neparvovec-rzyl (Luxturna^TM^)	NCT00999609	Phase III	Subretinal	Active, not recruiting	Spark Therapeutics	USA	31
*RPE65*	rAAV2-CB–hRPE65	NCT00749957	Phase I-II	Subretinal	Completed	Applied Genetic Technologies Corp	USA	12
*GUCY2D*	SAR439483	NCT03920007	Phase I–II	Subretinal	Active, not recruiting	Sanofi	USA	15
*CEP290*	QR-110	NCT03140969	Phase I/II	Intravitreal	Completed	ProQR Therapeutics	USA and Belgium	11
*CEP290*	AGN-151587	NCT03872479	Phase I–II	Subretinal	Recruiting	Allergan	USA	18
*RPE65* & *LRAT*	QLT091001	NCT01521793	Phase I	Oral	Completed	QLT Inc.	USA and Europe	27
*RPE65* & *LRAT*	QLT091001	NCT01014052	Phase I	Oral	Completed	QLT Inc.	USA and Europe	32
–	Human RPE cells	NCT03566147	Phase I	Subretinal	Unknown	Eyecure Therapeutics Inc.	China	30
–	Argus II retinal prosthesis	NCT01490827	Observational	Subretinal	Terminated	Second Sight Medical Products	Europe	52
–	RST-001	NCT02556736	Phase I/II	Intravitreal	Active, not recruiting	Allergan	USA	12
–	GS030-DP and GS030-MD	NCT03326336	Phase I/II	Intravitreal	Recruiting	GenSight Biologics	USA and Europe	18

EOSRD, early-onset severe retinal degeneration; LCA, leber congenital amaurosis; RPE, retinal pigment epithelium.

### General management of LCA/EOSRD

The current management for most patients with LCA/EOSRD remains symptomatic and supportive, with treatment of possible associated complications (cataract, vitreoretinal interphase abnormalities, cystoid macular oedema, keratoconus)^13 14^ and a recommendation for a balanced, healthy diet, rich in fruits and vegetables.^15^ Vitamin A, minerals and amino acids supplementation have failed to show a clear benefit for these patients, and are consequently not recommended.^16 17^ Ultraviolet (UV) light has been shown to cause oxidate stress in the retina, thus UV protection when outdoors is advised.^18^ Smoke and tobacco-free environments are also suggested, as there is evidence that smoking may further damage the retina.^19^ Patients benefit from correction of refractive error, use of low vision aids when possible and optimal access to educational and work-related opportunities. Infants with severe visual impairment may also have delays or difficulties with speech, social skills and behaviour, highlighting the importance of a multi-specialist approach.

Several clinical trials are currently available for individuals with inherited retinal degenerations (IRDs), including LCA/EOSRD, using different approaches. The optimal design of these studies is challenging. IRDs are rare, and therefore gathering large cohorts can be complicated; which is often addressed with multiple trial sites and consequently high costs. Also, determining the eligibility criteria and ideal therapeutic window may not be straightforward. Age is not always correlated with the severity of disease; the rate of degeneration differs significantly between individuals. Structural and functional retinal parameters are usually set as eligibility criteria, often based on natural history data. Robust prospective data take time to be gathered and there is rarely a consensus between different trials. The clinical trials currently available use different techniques as follows.

#### Genetic therapy

Given the relative immune privilege of the eye and its favourable anatomical characteristics, ophthalmology has been one of the areas in which genetic therapy has thrived,^20^ with IRD having been particularly targeted, partly due to its monogenic basis.

#### Gene supplementation

Gene supplementation is based on the delivery of DNA to the cells’ nuclei via a viral vector.^21^ This process leads to the transcription of a functional protein and is currently in trial in many autosomal recessive and X-linked conditions.^22^ Several LCA/EOSRD-associated genes have been targeted for this therapy.

#### *RPE65*-LCA/EOSRD

Retinal pigment epithelium-specific 65 kDa (*RPE65*, OMIM 180069) is an enzyme that catalyses a critical step in the visual cycle, within the RPE.^23^ Biallelic disease-causing variants in *RPE65* cause 5%–10% of all LCA/EOSRD cases and these patients show severe, early-onset night blindness.^1^ Clinical trials targeting replacement of *RPE65* have been in development since 2007.^24 25^

Bainbridge *et al* conducted a phase I/II trial in which twelve patients were treated with a recombinant adeno-associated virus 2 (rAAV2) containing *RPE65* complementary DNA, at two dose levels (NCT00643747).^26^ The surgery involved the administration of up to 1 mL of viral vector through a subretinal bleb involving the fovea.^25^ After a 3-year follow-up, six (50%) patients showed an improvement in retinal sensitivity (assessed by microperimetry and photopic and scotopic static perimetry), which was evident after 1–2 months of surgery, maintained over 6–12 months, and declining after this point, although remaining improved when compared with preintervention. Three patients (3/12, 25%) had intraocular inflammation that led to macular thinning and decreased acuity in two of them (2/12, 16.6%). The authors suggested a possible dose–response effect but did not identify a correlation between better response and younger age. The fact that the sensitivity declined after the first year led to the conclusion that the effect of this drug was temporary, variable and incomplete; and that an optimised, more potent therapy was required.^27^ This led to the phase I/II study NCT02781480, which involved the AAV5 vector OPTIRPE65 and 15 patients divided into three dose escalation groups and a paediatric expansion cohort. This drug was found to be safe, with most adverse events being transient and mild/moderate. Nine patients received the optimal concentration (1.0×10^11^ vg/mL) and all of them showed a statistically significant improvement in vision-guided mobility (maze navigation at different lux levels), retinal sensitivity (Octopus static perimetry), visual acuity, contrast sensitivity and reading speed, 6 months after the injection.^28^

Concomitantly, Hawswirth *et al* conducted a phase I/II trial in which a subretinal injection of rAAV2-CBSB-hRPE65 was performed in 15 young adults (NCT00481546).^29^ The cohort was divided into five subcohorts of different doses and volume of the viral vector, delivered in one or two injections. There were no reported serious adverse events (SAEs) and visual function improved in all patients to different degrees in the treated areas (measured by full-field stimulus testing (FST)) and remained stable for up to 36 months postinjection; although there was continued evidence of retinal degeneration.^30 31^

Concurrently, Spark Therapeutics was developing Voretigene neparvovec (Luxturna, NCT00999609). Luxturna is the first and only gene therapy for an IRD approved by the Food and Drug Administration (FDA, 2017).^32^ It is also the only treatment trial for *RPE65* that reached phase III. The latter involved 20 patients aged 3 and older, who had bilateral treatment with an AAV2 vector, with a 6 to 18 day interval between eyes.^33^ Primary endpoint was change in performance on a multiluminance mobility test, 1 year after the injection. A statistically significant difference was observed between the treated and control groups (p<0.05), with mild ocular adverse events related to the surgical procedure (transient ocular inflammation, elevated intraocular pressure and intraoperative retinal tears). After the approval from FDA, health insurances and healthcare systems are starting to cover this treatment for eligible patients.^34 35^ The National Health Service of England funds the use of Luxturna in patients who have two likely disease-causing variants in *RPE65* and remaining viable retinal cells. An ophthalmologist has to evaluate the child to assess their fitness to participate and refer them to one of the four treatment centres in the UK (https://retinauk.org.uk/information-support/luxturna/). This will lead to more patients and longer follow-up, which will enable a better understanding of the durability of treatment response. The longest follow-up published so far has been 3 years, where the efficacy appeared relatively well maintained.^36^ Patients between 4 and 44 years old have been treated and followed for up to 10 years.^37^

Weleber *et al* conducted a phase I/II trial where they treated twelve patients with rAAV2-CB-hRPE65 (NCT00749957). The participants were divided into two groups and received two different doses.^38^ No SAEs were reported, and visual function improved in 9 of 12 patients, as per best-corrected visual acuity (BCVA), visual field testing or responses to a quality-of-life questionnaire. The greatest improvement in BCVA was observed in the younger patients with better baseline BCVA.^38^

#### *GUCY2D*-LCA/EOSRD

Biallelic variants in *GUCY2D* account for 10%–20% of cases worldwide.^1^ *GUCY2D* (OMIM 600179) encodes the enzyme guanylate cyclase 2D, which synthesises cGMP, the intracellular messenger of photoreceptor stimulation.^39^ While the majority of disease-causing variants are associated with LCA (88%), a minority cause autosomal dominant cone dystrophy/cone-rod dystrophy (CRD).^40^ A genotype–phenotype correlation has been postulated, including missense variants being more commonly associated with CRD and null alleles with LCA.^40^ *GUCY2D*-LCA is characterised by severe congenital onset, profoundly reduced visual acuity, nystagmus, light sensitivity and absent/markedly reduced electroretinogram responses. However, it has been noted that these patients tend to show relatively preserved central photoreceptor structure until late in the disease.^41 42^ This feature makes *GUCY2D*-LCA particularly suitable for interventional gene therapy trials.

Boye *et al* have developed SAR439483, using a modified AAV capsid (Y733F), a subretinal gene therapy aiming to restore expression of guanylate cyclase 2D and thereby preserve the structure of rods and cones.^43^ It was found to be effective in knock-out mice, with improvement in electroretinography (ERG) responses.^44 45^ A phase I/II trial (NCT03920007) is on-going to enrol 15 participants; 9 in a dose escalation phase, and 6 in the dose expansion phase.^46^

#### *AIPL1*-LCA

Aryl hydrocarbon receptor-interacting protein-like 1 (AIPL1, OMIM 604392)-LCA is a rare cause of LCA (1%–2% of cases).^47 48^ This gene is expressed in photoreceptors and has a role in the synthesis of cGMP phosphodiesterase, a key element in phototransduction. Individuals with a damaging variant in *AIPL1* generally do not have residual outer retinal structure beyond the age of 4 years.^49^ A compassionate use gene therapy study is ongoing for *AIPL1*-LCA, in infants and young children with remaining outer retinal layers at the central macula. This consists of a unilateral subretinal injection of an AAV8 vector, and is taking place at Great Ormond Street Hospital and Moorfields Eye Hospital, London, UK.

### Gene editing and post-transcriptional regulation

Clustered regularly interspaced short palindromic repeats (CRISPR) and their associated enzyme (Cas) play a role in prokaryotic adaptive immunity.^50^ This system works as a physiological genome editing tool that cuts and deletes exogenous DNA, such as from viruses. Their discovery has led to their implementation as a tool for gene editing, targeting cardiovascular, neurological and neoplastic disorders, with previously no available treatment.^51^

Another method to suppress the expression of a damaging variant is through the use of small molecules designed to interact with pre-mRNA, known as antisense oligonucleotides (AON). Through this bonding, these molecules can modulate the pre-mRNA splicing (eg, attaching to an exon, they can block its transcription), or cause the degradation of certain transcripts.^52^ This technique is particularly useful for frameshift variants that cause the inclusion of pseudoexons.

#### *CEP290*-LCA/EOSRD

This gene (OMIM 610142) encodes a centrosomal protein of 290 kDa, a key component of cilia.^53^ As with other ciliopathies, damaging variants in this gene can present as an isolated ophthalmic condition, or as a syndromic disorder with kidney, skeletal and multiorgan pathologies. Disease-causing variants in *CEP290* have been associated with LCA, Joubert, Senior-Loken and Meckel syndromes.^54^ The intronic variant c.2991+1655A>G, p.(Cys998X) is the most frequent in LCA, with 60% to 90% of affected individuals carrying at least one c.2991+1655A>G allele.^55^ Patients with this variant rarely have extraocular manifestations. In vitro studies have shown that it creates a cryptic splice donor site resulting in the inclusion of an aberrant exon and a premature stop codon.^56^ This leads to a decreased amount of wildtype protein, which affects cilia function.

The first interventional phase I/II clinical trial treating individuals with *CEP290-*related LCA (NCT03140969) is targeting those carrying the variant c.2991+1655A>G in a homozygous or compound heterozygous state.^57^ The tested drug (QR-110) is an AON developed to block the aberrant splicing event and restore normal splicing. Ten patients were enroled and received a unilateral intravitreal injection of QR-110 every 3 months for up to 1 year.^58^ The study was deemed to be safe and no SAEs were reported. BCVA, macular optical coherence tomography (OCT) structure, mobility, nystagmus measurements and FST were selected as outcome measures.^59^ Improvement and/or stability was observed in all parameters in the treated eyes, after a follow-up period of at least 3 months after surgery. This study is now recruiting for phase III (NCT03913143).

The first CRISPR-mediated retinal gene therapy clinical trial (NCT03872479) using EDIT-101 is ongoing. This phase I/II study started in 2019 and aims to enrol 18 participants harbouring the c.2991+1655A>G variant. The study consists of a subretinal injection of an AAV5 vector containing *Staphylococcus aureus* Cas9 and *CEP290*-specific guide RNAs.^60 61^ This was tested in mice and non-human primates, with no SAEs reported. Furthermore, the Cas9 expression was noted to be restricted to photoreceptors and the CEP290 gene-editing rates correlated with the levels of both Cas9 mRNA and gRNA.^61^

### Pharmacotherapy

Visual cycle modulators have been under development for the last 10 years, with the aim of either decreasing the accumulation of various retinoid derivatives or to supply deficient compounds.^62^ In *RPE65*-related and *LRAT* (OMIM 604863)-related LCA/EOSRD, all-trans-retinal does not get converted back to 11-*cis*-retinal, interfering with the visual cycle, and ultimately leading to retinal degeneration and visual loss.^63^ It has been suggested that exogenous supply of 11-*cis*-retinal may prevent (or slow) photoreceptor degeneration.

A few studies (NCT01014052, NCT01521793) have tested the effect of supplementing 9-*cis-*retinyl acetate orally (QLT091001), in patients with *LRAT* and *RPE65*-related retinal degeneration. The first study involved a daily dose of 40 mg/m^2^ for 7 days, was completed in 2013 and included 18 participants.^64^ No SAEs were reported, and mild AEs were transient. An improvement in visual function was noted in 44% of participants with respect to visual field (20% expansion in kinetic perimetry documented at two consecutive study visits starting within 2 months of treatment) and in 67% for BCVA (≥5 Early Treatment Diabetic Retinopathy Study (ETDRS) letter score increase). A correlation between the thickness of the outer segments and the responsiveness to treatment was reported, suggesting that this structural parameter may predict the efficacy of the intervention.^65^ After a 2-year follow-up, 11 patients had returned to their baseline visual field and 10 to their baseline BCVA letter scores.^66^ A second trial tested the effect of repeated courses of this drug, in patients who had already received the first dose. Subjects received up to three more courses of once-daily oral QLT091001 (40 or 60 mg/m^2^) for 7 days, with a minimum of 3 weeks in between treatment courses.^67^ This study included 27 participants from various locations worldwide, with results pending. The effects of QLT091001 have also been tested in adults with delayed dark adaptation secondary to age-related macular dystrophy (NCT01999764) and in patients with *RPE65*-related autosomal dominant retinitis pigmentosa (RP, NCT01543906). The latter study showed an improvement in visual field in three out of five patients and in BCVA in one patient.^68^

### Photoreceptor and RPE transplantation

Patient-derived somatic cells can be reprogrammed to induced pluripotent stem cells (iPSCs), retinal precursor cells and specialised cells such as photoreceptors or RPE.^69 70^ This process aims to replace dystrophic photoreceptors and provide a trophic and metabolic structure to prevent their degeneration.^71 72^ Given this an autologous transplant, it has a relatively low risk of rejection and would likely not require long-term immunosuppression. Although the first animal studies suggested successful integration of the transplant into the host retina, more recent studies have identified that a cytoplasmic transfer of RNA and proteins can occur between donor and host tissue, instead of integration.^73 74^ Nevertheless, work is on-going with hopes of human clinical trials in the near future.

Subretinal transplantation of human embryonic stem cell derived RPE cells has been undertaken in individuals with age-related macular degeneration (AMD) and Stargardt macular dystrophy.^75 76^ No adverse proliferation, rejection or other safety issue related to the transplant were reported. After a 1 year follow-up, the investigators mentioned an improvement in BCVA in more than half of treated eyes. Increased subretinal pigmentation was also noted in 13 of 18 patients, although this was not correlated with improved visual function. iPSC–RPE cells have also been transplanted into the subretinal space as patches on biodegradable scaffolds, in animal models resembling AMD.^77^ Sharma *et al* reported improved integration and functionality of the RPE in patches, when compared with RPE cells in suspension. These cell therapies are now being attempted for the first time in patients with RP and LCA; including plans for *MERTK*-associated LCA.

### Optoelectronic devices

An optoelectronic device is able to transform an electrical signal to an optical one, and vice versa.^78^ There are different types regarding where they are located; epiretinal, subretinal, suprachoroidal and cortical implants are currently available or under evaluation.^79^ In the case of the retinal prostheses, their concept is to stimulate the preserved inner retinal layers by the implantation of an electronic device that would compensate for the outer retina not functioning properly.^80^ These are all composed of an external camera that capture light stimuli, a video processor that converts them into electric currents and a stimulator chip that directs them onto the retinal neuronal network. Some devices have been studied mainly for AMD (such as IRIS), whereas others target retinal and/or optic nerve degenerations.^79^

Argus II (Second Sight Medical Products) is the first retinal prosthetic device that became FDA approved, in 2013. It remains the only FDA approved retinal prosthesis and has been widely used, with over 350 implanted patients worldwide.^81^ Its production has been currently suspended, due to the company focusing on the development of new devices.^82^ A 5-year follow-up of 30 patients showed 24 SAEs among 12 patients (conjunctival erosion and dehiscence, hypotony, presumed endophthalmitis and retack of the device were the most common; corneal melt and retinal detachment were among the severest), treated with standard ophthalmic approaches. Three patients had the device completely or partially explanted due to associated complications, and two experienced device failure but were left implanted.^83^ This group also described an improvement in visual function, assessed by visual tasks such as following a high contrast line or spotting a door. This device is intended for individuals with advanced RP with a history of useful vision.^84^ However, in one of their clinical trials (NCT01490827), participants with severe to profound outer retinal degeneration were included; thereby patients with LCA may also have been included. Despite the improvements in visual function, as well as in quality of life assessments, limitations such as a field of view dependent on head position and limited electrodes translating into limited field remain to be addressed.^85^

## Optogenetics

The field of optogenetics aims to repurpose second-order and third-order neurons of the visual pathway (bipolar and ganglion cells),^86^ to replace the degenerated or misfunctioning photoreceptors.^87^ To do so, a gene encoding a light-sensitive opsin-based protein is introduced in these cells, photosensitising them.^88^ This approach is particularly useful due to being independent of the causative gene, and therefore suitable for a broad range of patients with IRDs. It also takes advantage of the usually preserved and functioning inner retinal layers. The feasibility of this strategy has been tested in rats and mice, where they showed a positive impact measured by restored visual evoked potentials and optomotor responses.^89–91^

Due to favourable preclinical data, there are currently two trials involving optogenetics’ techniques in patients with advanced RP. We believe that patients with LCA may be eligible for these trials as well, given that they meet the requirement of severely decreased visual acuity and retinal degeneration. NCT02556736 is a phase I/IIa, open label, dose-escalation study launched in 2015 at multiple locations in the USA. It has fully recruited 12 adults who have received an intravitreal injection of the drug RST-001, a gene therapy encoding the protein channelrhodopsin-2.^92^ The second trial, NCT03326336, launched in 2017, is still recruiting in the USA, Europe and UK. It is a phase I/II study that combines a gene therapy called GS030-DP (rAAV2 vector containing the optimised channelrhodopsin ChrimsonR-tdTomato, delivered intravitreally) and stimulating glasses named GS030-MD. It will involve 18 participants divided into three dose-escalating cohorts and one extension cohort of three more individuals. Results are still pending for both studies.

## Conclusions

Undoubtfully, tremendous progress has been made since the first description of LCA by Theodore Leber 150 years ago. In depth multimodal evaluation and genotypic characterisation have made this condition attractive for pioneering research that could improve patients’ (and their families) quality of life, and advance the frontiers of medicine. Outstanding research has led to this condition being the only IRD with an FDA-approved gene therapy, and with 29 registered clinical trials, 19 of which are interventional (www.clinicaltrials.gov, accessed on October 2020).

In this review, we have focused on recent and ongoing clinical trials with the greatest likelihood of successfully treating LCA/EOSRD. A wide range of different approaches and interventions are in clinical evaluation, with many more in late preclinical stages, hoping to treat conditions caused by variants in other genes, as well as applying different techniques to hopefully obtain better outcomes.^93–96^

There is great optimism (that we should share with our patients) that over the next 5 years, clinical trials for LCA/EOSRD will continue to increase in number, with more treatments being approved. More genes and more treatment approaches are in the pipeline and will continue their development to a clinical trial and, hopefully, an approved drug in the next few years. There are many challenges, including establishing these therapies to be cost effective and scalable—including if more genetic therapies could be delivered intravitreally compared with subretinally, with all the inherent benefits. In addition, should durability wane, it will need to be determined if and when to retreat individuals that have already undergone gene therapy. A further future area of investigation will be the pros and cons of a patient receiving different treatments both in the same or in the contralateral eye, at the same or subsequent timepoints. As more therapies are developed, research exploring their synergic (or antagonistic) effect(s) will need to be undertaken.
